# Factors affecting adherence to antiretroviral therapy among children and adolescents living with HIV in the Mbita Sub-County Hospital, Homa Bay- Kenya

**DOI:** 10.4314/ahs.v21i1.4S

**Published:** 2021-05

**Authors:** William N Tanyi, Onesmus Gachuno, Theresa Odero, Carey Farquhar, David Kimosop, Allan Mayi

**Affiliations:** 1 Afya Bora Consortium Fellowship on Global Health Leadership, Department of Global Health, University of Washington, Seattle- USA; 2 Elizabeth Glaser Pediatric AIDS Foundation (EGPAF), Nairobi- Kenya; 3 Centers for Disease Control and Prevention (CDC), Kenya; 4 President's Emergency Program for AIDS Relief (PEPFAR), Kenya; 5 Ministry of Health (MOH), Homa Bay, Kenya; 6 Kenyatta National Hospital, Nairobi, Kenya; 7 The National AIDS and STI Control Program (NASCOP), Nairobi, Kenya; 8 Sub County Hospital, Mbita, Kenya

**Keywords:** Adherence, clinic attendance, antiretroviral therapy, HIV, virologic suppression

## Abstract

**Background:**

Adequate adherence to antiretroviral therapy (ART) is key to the successful treatment of children and adolescents living with HIV. Continuous ART Adherence is the key factor for virologic suppression and stability of the immune system and prevents the occurrence of opportunistic infections. Children and adolescents struggle with adherence to ART for various reasons, including a poor psychosocial support system and clinic attendance.

**Objectives:**

To describe the uptake of HIV treatment services among children and adolescents in the Mbita Sub-County Hospital, Homa Bay and determine how schooling, clinic attendance, and type of pill/regimen affect adherence to ART and viral suppression.

**Methods:**

This retrospective study was conducted at the Mbita Sub-County Hospital. Medical chart data was abstracted from the hospital files of children and adolescents between the ages of 0–19 years on antiretroviral therapy, between the periods of October 2016 and September, 2017. Data was analyzed using measures of central tendency, and cross-tabulations were done to compare schooling, clinic attendance, type of pill/regimen and viral suppression. Univariate and multivariate logistic regression analyses were conducted to determine associations between groups.

**Results:**

According to patient files reviewed, majority of patients, 244(91.4%) were enrolled into care within 2 weeks of HIV diagnosis according to guidelines, and 193(73.1 %) remained enrolled in care at end of study period. An overall viral suppression of 74.2 %( 132) was recorded. Of all the files reviewed, 121(74.7%) of patients attending school suppressed against 11(68.8 %) out of school, p=0.280. Suppression among Day and boarding reported at 78.6 %( 11) and 74.8 %( 113) of those out of school, respectively, p=0.533. Participants in primary school, 17(85.0%) suppressed better than those in secondary school, 102(73.4%), p=0.263. Keeping clinic appointments among eligible patient files reviewed decreased from 83.1% at 3 months, p=0.016, to 76.6%, p=0.526 at 6 months and to 52.9% at 12 months, p=0.278. Only 3- month clinic appointment return rates and Enhanced Adherence Counseling (EAC) were significant predictors of viral supression χ^2^ (2) = 0.280, p = 0.869 (> 0.05).

**Conclusion:**

The clinic attendance rate within the first 3 months, and Enhanced Adherence Counseling (EAC) were significant predictors of viral suppression, and therefore adherence to antiretroviral therapy.

## Introduction

According to the UNAIDS Global Report on the global AIDS epidemic (2013), approximately 36.7 million people worldwide are living with HIV/AIDS, and of these 2.1 million are children 15 years old and younger, accounting for 6% of all people living with HIV/AIDS. Also, children account for 9% of all new HIV infections, and 11% of all AIDS-related deaths globally. A vast majority (1.7 million or 84%) of these children live in sub-Saharan Africa, and less than half (43%) are on antiretroviral therapy (ART). In Kenya, approximate 6,613 new HIV infections occur in children yearly, and this accounts for 15.1% of all new infections in the country (KHCP, 2014). Homa Bay has an HIV prevalence of 26% (4.5 times above the national average for Kenya), according to the Kenya HIV County Profiles (KHCP, 2014) and 28% of all new infections in the County occur in adolescents. Of the 7,945 children currently in care in Homa Bay, 7,418 are on ART, and barely 40% of them have achieved viral suppression. Children under 1 year of age are among the most vulnerable to HIV, and evidence shows that early initiation of ART in infants with HIV can save lives, yet coverage of critical intervention among children remains low (KHCP, 2014).

The National AIDS and STI Control Program (NASCOP) Guidelines (2016) on the Use of Antiretroviral Drugs for Treating and Preventing HIV Infection in Kenya recommend that all people living with HIV (PLHIV) must benefit from ART, and ART should be initiated as soon as the patient was ready to start, preferably within two weeks from the time of HIV diagnosis. Adequate adherence to ART is key to successful treatment of PLHIV with a corresponding viral suppression, improved health outcomes and significant improvement in life expectancy (NASCOP, 2016).

The World Health Organization (WHO) defines adherence as “the extent to which a person's behavior- taking medication, following a diet, and/or executing lifestyle changes, corresponds with agreed recommendations from a healthcare provider”. In order to achieve virologic suppression, patients are required to maintain more than 90–95% adherence to antiretroviral therapy (ART) (Kim, Gerver, Fidler, & Ward, 2014). Unfortunately, children and adolescents accessing ART usually face unique challenges stemming from their youthful age, family settings, and environment (Merzel & Van-Devanter, 2008). For example, cognitive deficits, parental illness, depression, or behavioral issues may adversely affect their adherence to ART, leading to early ART failure, development of resistance to treatment, and subsequent reduction in treatment options.

Homa Bay County has the highest prevalence (26%) of HIV in Kenya, with only a 40% suppression rates for children and adolescents. Adequate adherence to ART is key to successful treatment with a corresponding viral suppression and improved health outcomes among these children and adolescents living with HIV. This requires continuous assessment of barriers to adherence to enable adequate support and achievement of viral suppression. Adherence in Homa Bay health care facilities is assessed through patient self-reporting, and this is a subjective and unreliable method of assessment, with oftentimes overestimated figures. Children and adolescents are challenged to effectively suppress the virus when compared to adults in the majority of facilities in Homa Bay, even within the same facility, and this requires an understanding of the factors leading to this discrepancy.

The aim of this study was to describe the uptake of HIV treatment services among children and adolescents in the Mbita Sub-county hospital, Homa Bay. We wanted to describe how schooling, clinic attendance and type of pill/regimen affected adherence to ART and viral suppression. Instead of self-report, we will provide an objective framework for measuring adherence to ART through viral suppression as the outcome variable, as well as highlight strategies of improving adherence to ART among HIV+ children and adolescents in Mbita Sub-county.

## Methods

### Study Design

This was a retrospective study conducted at the Mbita Sub-county hospital in Homa Bay, Kenya. Data abstraction was done from the hospital medical records of eligible patients who accessed care between the periods of October, 2016 to September, 2017. Approval to carry out the study was sought from Kenyatta National Hospital Ethical Review Committee (KNH ERC), Chesapeake, and the CDC, and a waiver of consent was obtained to proceed with the study.

### Study Site

The Mbita Sub-county hospital is the coordinating and referral center for smaller health care units within the Sub-county, and serves a catchment population of approximately 7,249 people in the Homa Bay County of Kenya. The hospital receives patients through the Out Patient Department (OPD), Maternal and Child Health (MCH) Clinic, HIV Care Clinic, TB Clinic, Voluntary Male Medical Circumcision (VMMC) Clinic and the HIV Testing Services (HTS) Clinic.

### Study Population

Two hundred and sixty-four (N=264) children and adolescents were found to be eligible for the study following a query of the OpenMRS database that was done. The records were eligible for review if the participants were between the ages of 0–19 years, HIV positive, on ART prior to study period, and accessed HIV care at the Mbita Sub-county hospital during the study period. Exclusion criteria were older than 19 years, HIV negative, and accessed care elsewhere.

### Data Abstraction/Tools

#### Tools

For the purpose of this study, Viral Load (VL) measurement was used as the biomarker for measuring adherence to ART. A VL< 1000copies/ml was considered confirmation of adherence (NASCOP, 2016). Whereas a VL > 1000 copies/ml was considered poor adherence to ART.

#### Data Storage

All data collected was stored on a password-protected computer monitored by the researcher, and all patient identifiers coded for confidentiality purposes.

### Data Analysis

Data were imported into SPSS, version 2014 and were examined for outliers and missing results. Descriptive analysis was done for the variables of interest. The outcome variables, i.e. viral load suppression and clinic appointments were stratified with the clinic and demographic variables. The clinic variables included adherence using Morisky forms, viral suppression, opportunistic infections, nutritional support, booster and EAC adherence, and disclosure. The demographic variables included gender, age, parent accompaniment and school attendance. Where applicable, the Pearson Chisquare and Fisher's exact tests were used to ascertain association amongst the clinical variables and p-values were calculated. A P value <0.05 was considered statistically significant.

## Results

### Participant Demographics

Table 1 shows a distribution of participants according to demographics.

**Table 1:** Distribution of Participant Demographics

	Age (years)				Totals N=264
	0–4 N=34	5 – 9 N=64	10 – 14 N=84	15 – 19 N=82	
**Gender**					
Male	16 (13.4%)	39 (32.8%)	45 (37.8%)	19 (16%)	119 (45.1%)
Female	18 (12.4%)	25 (17.2%)	39 (26.9%)	63 (43.5%)	145 (54.9%)
**School Attendance**					
Yes	22 (9.2%)	64 (26.9%)	84 (35.3%)	68 (28.6%)	238 (90.2%)
No	12 (46.2%)	0 (0%)	0 (0%)	14 (53.8%)	26 (9.8%)
**Patient Outcomes**					
Alive	17 (8.8%)	55 (28.5%)	71 (36.8%)	50 (25.9%)	193 (73.1%)
Dead	0 (0%)	0 (0%)	0 (0%)	2 (100%)	2 (0.8%)
Lost to follow up	7 (24.1%)	2 (6.9%)	4 (13.8%)	16 (55.2%)	29 (10.9%)
Transfer out	7 (17.5%)	7 (17.5%)	12 (30%)	14 (35%)	40 (15.2%)

### Factors affecting adherence

The study explored the associations between various patient factors and viral suppression using cross tabulations with results shown in Table 2 below.

**Table 2:** Distribution of Adherence Factors against Viral Suppression among Children and Adolescents

	Viral Suppression		Totals	χ^2^ (p-value)
	Yes	No		
**Gender**				
*Male*	69 (78.4%)	19 (21.6%)	88 (49.4%)	**1.642 (0.200)**
*Female*	63 (70%)	27 (30%)	90 (50.6%)	
**Age**				
*Children 0–9*	56 (81.2%)	13 (18.8%)	69 (38.8%)	**2.883 (0.090)**
*Adolescents 10–19*	76 (69.7%)	33 (30.3%)	109 (61.2%)	
**Attending School**				
*No*	11 (68.8%)	5 (31.2%)	16 (8.9%)	**1.165 (0.280)**
*Yes*	121 (74.7%)	41 (25.3%)	162 (91.1%)	
**School Type(subset of in school)**				
Boarding	113 (74.8%)	38 (25.2%)	151 (91.5%)	**1.259 (0.533)**
Day school	11 (78.6%)	3 (21.4%)	14 (8.5%)	
**Education Level *(subset of in school)***				
*Primary*	102 (73.4%)	37 (26.6%)	139 (87.4%)	**1.254 (0.263)**
Secondary	17 (85.0%)	3 (15.0%)	20 (12.6%)	
**Clinic Appointments at 3month**				
Kept clinic appointment	115 (77.7%)	33 (22.3%)	148 (83.1%)	**5.760 (0.016)**
Missed clinic appointment	17 (56.7%)	13 (43.3%)	30 (16.9%)	
**Clinic Appointments at 6 month**				
Kept clinic appointment	100 (76.3%)	31 (23.7%)	131 (76.6%)	**1.277 (0.528)**
Missed clinic appointment	27 (67.5%)	13 (32.5%)	40 (23.4%)	
**Clinic Appointments at 12 month**				
Kept clinic appointment	12 (66.7%)	6 (33.3%)	18 (52.9%)	**3.853 (0.278)**
Missed clinic appointment	9 (56.2%)	7 (43.8%)	16 (47.1%)	
**ART Adherence Morisky Good**				
Yes	126 (75.4%)	41 (24.6%)	167 (93.8%)	**16.584 (<0.001)**
Not done	6 (54.5%)	5 (45.5%)	11 (6.2%)	
**Received Booster Adherence**				
No	13 (36.1%)	23 (63.9%)	36 (20.2%)	**60.158 (<0.001)**
Yes	119 (83.8%)	23 (16.2%)	142 (79.8%)	
**Started on Isoniazid** **Prevention Therapy (IPT)**	132 (74.2%)	46 (25.8%)	178 (100%)	-
**On Nutritional Support**				
No	128 (74.4%)	44 (25.6%)	172 (96.6%)	**0.182 (0.670)**
Yes	4 (66.7%)	2 (33.3%)	6 (3.4%)	
**HIV Disclosure Done**				
Yes	111 (72.5%)	42 (27.5%)	153 (85.9%)	**1.926 (0.382)**
No	21 (84%)	4 (16%)	25 (14.1%)	
**Antiretroviral Pill Regimen**				
1^st^ line	101 (73.7%)	36 (26.3%)	137 (77.0%)	**0.059 (0.809)**
2^nd^ line	31 (75.6%)	10 (24.4%)	41 (23%)	
**Enhanced Adherence Counseling (EAC)**				
Yes	39 (88.6%)	5 (11.4%)	44 (89.8%)	**144.6 (<0.001)**
No	5 (100%)	0 (0%)	5 (10.2%)	

None of the school-related variables were associated with viral suppression based on school attendance, school type and education level with viral suppression ( χ^2^ (1) = 1.165, p = .280, χ^2^ (1) = 1.259, p = .533, χ^2^ (1) = 1.254, p = 0.263,) respectively.

A total of 167(93.8%) of participants were assessed with the ART Morisky scale, and adherence was reported as good for 126(75.4%) that were virally suppressed; while of the 142(79.8%) who needed Booster adherence sessions, 119(83.8%) were virally suppressed. The association between the Morisky adherence scale and viral suppression was statistically significant (χ^2^ (1)= 16.584, p < .001). Furthermore, booster adherence had a significant association with viral suppression (χ^2^ (2) = 60.158, p < .001). All participants of the study were placed on Isoniazid Prevention Therapy (IPT), with 132(74.2%) virally suppressed.

Of the 153(85.9%) participants who had an age-appropriate disclosure done, 111(72.5%) were virally suppressed. Also, patients on first line regimen were suppressing at about the same rate as those on second line, at a rate of 101(73.7%) and 31(75.6%) respectively. Out of a total of 49 files of patients eligible for Enhanced Adherence Counseling (EAC), 44(89.8%) patients successfully underwent an EAC, and of these 39(88.6%) were virally suppressed.

### Clinic appointment vs. viral suppression

Overally, clinic appointments decreased from 83.1% at 3 months, to 76.6% at 6 months and to 52.9% at 12 months.

#### At 3- months

Of the 148(83.1%) of participants who kept the 3-month appointment, 115(77.7%) were virally suppressed while 33(22.3%) were not. Of the 30(16.9%) who missed the 3-month appointment, 17(56.7%) were virally suppressed. The total viral suppression rate for the 3-month clinic appointment was 74.2%. The association between clinical appointment at 3 months and viral suppression was found to be significant (χ^2^ (1)= 5.760, p = .016), p-value of <0.05 said to be significant.

#### At 6- months appointment

A total of 131(76.6%) of participants kept the 6- month appointment, and 100(76.3%) were virally suppressed while 40(23.4%) missed the 6- months appointment, yet 27(67.5%) of them were virally suppressed. The total 6-months viral suppression rate was 71.3%.

#### At 12- months appointment

A total of 18(52.9%) of participants kept the 12-months appointment, with 12(66.7%) of them virally suppressed. 16(47.1%) of them did not keep the 12-month appointment, yet 9(56.2%) of those were virally suppressed. The total 12- months suppression rate was 61.7%.

### Logistic Regression

Results displayed in table 2 show that Booster adherence, Morisky assessment, EAC and keeping 3-month clinic appointments were significant, (P-values <0.05. These factors affecting viral suppression were further subjected to logistic regression with results shown on table 3 below. Only EAC and keeping 3-month appointment were observed to be significant.

**Table 3:** Logistic Regression Analysis for Significant Variables

Variable	B	S.E.	Wald	df	Sig.	Exp(B)	95% C.I. for EXP(B)	
EAC based on 1st Viral Load results			37.173	2	0.000			
EAC Done (1)	-25.878	17601.79	0.000	1	0.999	0.000	0.000	
EAC not Done (2)	-6.683	1.096	37.17	1	0.000	0.001	0.000	0.011
Kept 3_month appointment (1)	-2.267	1.244	3.32	1	0.068	0.104	0.009	1.187
Constant	4.914	1.022	23.126	1	0.000	136.213		

### Variable(s) entered on step 1

EAC done based on 1st VL results, 3_month appointment

### Model Summary

-2 Log likelihood = 47.677, Cox & Snell R Square = 0.583, Nagelkerke R Square = 0.856 Hosmer and Lemeshow Test: χ2 (2) = 0.280, p = 0.869 The findings from the logistic regression indicate that 3-month clinic appointment and EAC done were significant predictors of viral suppression χ^2^ (2) = 0.280, p = 0.869 (> 0.05). The two predictors explain 85.6% (Nagelkerke R Square) of the variability of viral suppression. While the overall EAC bsed on 1st viral load results were significant, Wald χ2 (2) = 37.173, p < 0.001, not doing EAC for those not virally suppressed based on the 1^st^ viral load resulted in significant decreases of the log-odds of viral suppression, -6.683, p < 0.001 (95% CI: 0.000 – 0.011).

## Discussion

In this study, viral suppression was used as the tool to measure adherence to ART among all the participants of the study. This value is much more objective and reliable than patient self-report tools such as the visual analogue scale (VAS), AIDS Clinical Trial Groups (ACTG) questionnaires and surveys, and the Morisky medication adherence scale (MMAS). Patient self-report tools are prone to bias such as social desirability and recall bias, anday report overestimated results for adherence when compared to a more objective tool as viral suppression, as used in this study.

Overall, of all the patient files reviewed for the study (N=264), 190(72.3%) remained actively engaged in care at the end of the study, while 41(15.4%) transferred out, 31(11.6%) were lost to follow-up, and 2(0.8%) were dead.

### Uptake of HIV treatment services

Mbita Sub-county hospital offered all patients various HIV care and treatment services during the period they accessed care at the facility, from time of HIV testing to linkage and retention, and subsequent follow-up clinic appointments. Two hundred and forty-four, 244(91.4%) were commenced on ART within two weeks of HIV diagnosis with a positive HIV test result, and this was much in line with NASCOP guidelines of commencing patients on ART within two weeks of HIV diagnosis. A good clinic appointment record is a predictor of adherence to ART and viral suppression across all age groups.

Disclosure of HIV status is predictive of good clinic attendance and eventually leads to good adherence to ART and viral suppression. All the adolescents in the study had achieved disclosure during the period of study, and this is a good indicator of their good viral suppression rates during the same period of study.

### Clinic attendance, schooling, and regimen/pill type

After adjusting for patients lost to follow-up, transferred out and dead, the sample size reduced to 193 (N= 193), and this is the number of patients that were active in care at the end of the study period. Children in the age group 0–9 years suppressed better than those in the age adolescent age group, and the former adhered better to treatment better than the latter. Keeping a good clinic attendance is therefore a predictor of good adherence to ART, and this finding is consistent with studies conducted elsewhere^6^. Patients who kept their clinic appointments had issues addressed on time before they became problematic, and therefore enjoyed booster adherence and Enhanced Adherence Counseling when the need arose, with the attendant result of viral suppression.

## Limitations

Key informant perspective was not obtained from health care providers, parents or caregivers, and patients themselves does not give a complete story of the factors that affect adherence in the Mbita Sub-county hospital, especially at the patient level. Also, the absence of tools such as the pill count and pharmacy refill records recommended by NASCOP to measure adherence was another limitation to this study.

This study found that clinic attendance at 3months, and Enhanced Adherence Counseling (EAC) are significant predictors of viral suppression, and therefore good adherence to antiretroviral therapy. Health care providers should build a robust psychosocial support system through parents, caregivers, medication buddies, within the first three months of ART initiation to help children and adolescents achieve viral suppression and stay virally suppressed. Upon detection of a high viral load, health care providers and adhesion counselors should reassess and support their patients through a booster adherence program with the ultimately goal of improved adherence and viral suppression.
